# Morphological, Physiological and Proteomic Analyses Provide Insights into the Improvement of Castor Bean Productivity of a Dwarf Variety in Comparing with a High-Stalk Variety

**DOI:** 10.3389/fpls.2016.01473

**Published:** 2016-09-29

**Authors:** Wenjun Hu, Lin Chen, Xiaoyun Qiu, Hongling Lu, Jia Wei, Yueqing Bai, Ningjia He, Rongbin Hu, Li Sun, Hong Zhang, Guoxin Shen

**Affiliations:** ^1^Zhejiang Academy of Agricultural SciencesHangzhou, China; ^2^State Key Laboratory of Silkworm Genome Biology, Southwest UniversityChongqing, China; ^3^Department of Biological Sciences, Texas Tech UniversityLubbock, TX, USA

**Keywords:** *Ricinus communis*, castor bean productivity, agriculture, photosynthesis, plant proteomics

## Abstract

*Ricinus communis* displays a broad range of phenotypic diversity in size, with dwarf, common, and large-sized varieties. To better understand the differences in plant productivity between a high-stalk variety and a dwarf variety under normal growth conditions, we carried out a comparative proteomic study between Zhebi 100 (a high stalk variety) and Zhebi 26 (a dwarf variety) combined with agronomic and physiological analyses. Over 1000 proteins were detected, 38 of which differed significantly between the two varieties and were identified by mass spectrometry. Compared with Zhebi 100, we found that photosynthesis, energy, and protein biosynthesis related proteins decreased in abundance in Zhebi 26. The lower yield of the dwarf castor is likely related to its lower photosynthetic rate, therefore we hypothesize that the lower yield of the dwarf castor, in comparing to high stalk castor, could be increased by increasing planting density. Consequently, we demonstrated that at the higher planting density in Zhebi 26 (36,000 seedlings/hm^2^) can achieve a higher yield than that of Zhebi 100 (12,000 seedlings/hm^2^). Proteomic and physiological studies showed that for developing dwarf *R. communis* cultivar that is suitable for large scale-production (i.e., mechanical harvesting), it is imperative to identify the optimum planting density that will contribute to higher leaf area index, higher photosynthesis, and eventually higher productivity.

## Introduction

The castor bean (*Ricinus communis*) is a tropical perennial shrub and field crop originated in Africa, but has now been introduced worldwide and is widely cultivated (Chan et al., 2010). It can be cross- and self-pollinated, and studies have revealed low levels of genetic variation among castor bean germplasm worldwide (Allan et al., 2008; Foster et al., 2010). Because of the nearly uniform ricinoleic acid content of castor oil and its unique fatty acid properties, castor beans are a valuable oilseed crop for the cosmetics industry, specialty lubricants, and biomedical and specialty chemical applications. *R. communis* has also been proposed as a potential source of biodiesel feedstock because of its high seed oil content (Da Silva et al., 2006), and the ease with which it can be cultivated in unfavorable crop-growing environments has contributed to its appeal as a crop in tropical developing nations. Furthermore, the castor plant is commonly cultivated in many counties for its leaves to feed the Eri silkworm (*Attacus Cynthia ricini* Boisduval), which provides Eri silk, a high-quality natural protein fiber.

*R. communis* is produced in about 30 countries for commercial purposes, among which India, China and Brazil account for >90% of the world's production (Severino et al., 2012). Mechanized castor production is possible and needs to become mandatory to sustain or increase global castor production. Because the three main countries producing castor are experiencing rapid economic and social development, the labor required for traditional castor production has become expensive (Severino et al., 2012). Currently, only limited areas of castor production are fully mechanized because of the lack of dwarf-internode and commercial cultivars (Baldanzi et al., 2003). Therefore, the main challenge in developing new cultivars is the adaptation of castor plants to mechanical harvesting. The development of appropriate new castor cultivars should be enhanced by improved knowledge of the genetics and molecular biology of the species. Mutation breeding has shown that irradiation of castor seeds and seedlings produces mutants with desirable characteristics including semi-dwarfs with higher yield potential and earlier maturity (Sujatha et al., 2008).

*R. communis* has a complex genetic background, which causes difficulties in genomic mutation and gene cloning. Transcriptome and gene expression analyses via measurement of mRNA levels have contributed greatly to characterizing mutants of rice and *Arabidopsis*. In contrast, there are obstacles to studying gene expression in *R. communis*. Moreover, levels of mRNAs, the key players in the cell, measured in qualitative terms do not always correlate well with phenotypes because of post-transcriptional regulation mechanisms. Therefore, proteome studies aimed at the complete set of genome-encoded proteins may complement the shortcoming of transcriptome approaches. Here, we present a study to identify the differences between a dwarf castor variety and a high stalk variety by using an integrated approach of proteomics, physiological analysis, and field experiments.

Dwarfism was an attractive phenotype of the “Green Revolution” and is still a desirable agronomic trait for crop cultivators as it is associated with high yield potential, improved lodging resistance, and higher fertility (Li et al., 2010). Since 1960s, new varieties of grain crops with short stems and substantially improved yields were developed (Conway and Toenniessen, 1999). Castor is a tropical plant that can be as short as two feet in height, or as tall as a moderate-sized tree. The development of improved varieties using molecular technologies will help ensure farmers across the world enjoy the economic potential of this crop (Auld et al., 2010). In this study, our main objective was to identify candidate genes and proteins that can be used to create a dwarf castor cultivar with high yield for agricultural applications and mechanical harvesting.

The specific aim of our research was to identify the differential protein changed in abundance comparing Zhebi 100 (a high stalk variety, 12,000 seedlings/hm^2^) and Zhebi 26 (a dwarf variety, 36,000 seedlings/hm^2^) in the highest yields, and find the relationship between castor yield and photosynthesis. Furthermore, we tested our hypothesis that increasing the effective photosynthetic area through increased planting density could make up for the low photosynthetic capacity of dwarf varieties.

## Materials and methods

### Plant materials

The experimental castor varieties, Zhebi 100 and Zhebi 26, were introduced from South Africa. In field experiments (Hangzhou, China; 120°12′ E, 30°16′ N, altitude 20–60 m), castor seeds were sown on April 15 during 2012–2015 with three replicates. The field experiment was based on complete randomized block design with three replicates at five different planting densities, i.e., 8000 seedlings/hm^2^, 12,000 seedlings/hm^2^, 24,000 seedlings/hm^2^, 36,000 seedlings/hm^2^, and 48,000 seedlings/hm^2^. Each replicate plot was 30 m^2^. For Zhebi 100, the plant distance was 100 cm, and the row spacing was 125, 83, 42, 28, and 21 cm, respectively. For Zhebi 26, the plant distance was 50 cm, and the row spacing was 250, 166, 84, 56, and 42 cm, respectively. Field management is carried out according to farmer's practice.

Ninety days after planting, six plants from each variety were randomly selected, and their morphological traits and photosynthetic parameters were measured. Mature leaves were taken from field *R. communis* plants in the experimental field. The samples were located at the same leaf position and orientation and were taken on the same day and hour during the active growth season (summer). After 90 days of growth, mature leaves and other plant tissues were selected for physiological measurements and protein extraction. Leaves were washed *in situ* with tap water, dried with filter paper, and then frozen in liquid nitrogen immediately. Samples were stored at −80°C until protein extractions were done.

### Physiological index measurements

Seedling performance was assessed in terms of biomass, separated into roots, stems, leaves, flowers, and seeds under 12,000 seedlings/hm^2^ in Zhebi 100, 36,000 seedlings/hm^2^ in Zhebi 26, respectively. Plant materials were oven-dried (70°C) to constant weight. A mean value was obtained from 10 seedlings. Each treatment contained three biological replicates. We used the average of the four replicates of 10 seedlings as the mean value of seedling weight.

*R. communis* leaves (0.1 g of fresh weight) were prepared, and chlorophyll was extracted with ice-cold 80% v/v acetone. Absorption of the extract was measured at 663 and 646 nm with a spectrometer (Varian Cary 50 UV-VIS) and chlorophyll content was calculated with formulae proposed by Wellburn (1994). The formulae used were as follows:

$$\begin{array}{l} {\text{C}}_{\text{a}}=11.74\;{\text{A}}_{663.8}-2.66\;{\text{A}}_{646.8};{\text{C}}_{\text{b}}=22.91\;{\text{A}}_{646.8}-4.53\;{\text{A}}_{663.8} \end{array}$$

Eight seedlings per castor variety were randomly selected for net photosynthetic rate (Pn) and chlorophyll fluorescence measurements. Pn was measured using a portable photosynthesis system (LI-6400, Li-Cor Inc., Lincoln, NE, USA), as described previously (Wei et al., 2015). The leaf area was measured using a leaf area meter of LI-3100 (Li-Cor Inc., Lincoln, Nebraska, USA). Leaf area index (LAI) was calculated as a ratio of the leaf area from a given land area to land area. According to the method of Chen et al. (2013), leaf chlorophyll fluorescence was measured using a pulse-amplitude-modulation fluorometer (PAM-2100, Heinz Walz, Effeltrich, Germany). The seedlings were exposed to sunlight for at least 15 min prior to Pn measurement. For each seedling, at least five measurements were made. Mean values were obtained from 8 replicates.

### Protein extraction

For Zhebi 100, the planting distance was 100 cm, and the row spacing was 83 cm, planting density is 12,000 seedlings/hm^2^. For Zhebi 26, the planting distance was 50 cm, and the row spacing was 56 cm, planting density is 36,000 seedlings/hm^2^. Leaves from these treatments were used for proteomics and Western blot analyses. Total *R. communis* leaf protein extraction was performed using the phenol extraction method (Hu et al., 2016), with slight modifications. Briefly, frozen *R. communis* leaves (1.0 g) were ground using a mortar and pestle in liquid nitrogen to a fine powder with an equal amount of polyvinyl polypyrrolidone. Next, the ground powder was homogenized in pre-cooled protein extraction buffer (20 mM Tris-HCl pH 7.5, 250 mM sucrose, 10 mM ethylene diamine tetraacetic acid, 1% Triton X-100, 1 mM 1,4-dithiothreitol and 1 mM phenylmethyl-sulfonyl fluoride) on ice. The pellets were then washed with ice-cold acetone for three times. The final washed protein pellets were dried by vacuum centrifugation to remove any remaining acetone and dissolved in lysis buffer (8 M urea, 2 M thiourea, 1% DTT, 4% CHAPS, 0.5% IPG buffer pH 4–7). The protein concentrations of the lysates were determined using a 2-D Quant Kit (GE Healthcare Amersham Bioscience, Little Chalfont, UK).

### Two-dimensional electrophoresis and data analysis

Two-dimensional electrophoresis (2-DE) was conducted according to Hu et al. (2014c). The protein samples (500 μg) were loaded onto Immobiline DryStrips (18 cm long, pH 4–7, GE Healthcare) during the rehydration step at room temperature for 12 h. Isoelectric focusing (IEF) was performed using an Ettan IPGphor isoelectric focusing system (GE Healthcare) as follows: 300 V for 1 h, 500 V for 1 h, 1000 V for 1 h, a gradient to 8000 V for 4 h, and kept at 8000 V for a total of 80,000 volt-hours (Vh) at 20°C. After IEF, the focused strips were equilibrated in equilibration buffer as described by Hu et al. (2014a). For the second dimension electrophoresis, the proteins were separated on 15% SDS polyacrylamide gels. Subsequently, the gels were stained using Coomassie Brilliant Blue (CBB) R-250. The 2-DE gel images were acquired with an image scanner (Uniscan M3600, China) and analyzed using the PDQuest software (Version 8.01, Bio-Rad, Hercules, CA, USA). Protein spots with significant (>2-fold change) and reproducible changes were selected for MS analysis.

### Identification of proteins and classification

Protein digestion and identification were performed according to Hu et al. (2014c). Protein spots were identified using a 4800 Plus MALDI-TOF/TOF Proteomics Analyzer (Applied Biosystems, USA). Tryptic peptide masses were searched according to the corresponding annotations in the National Center for Biotechnology Information non-redundant (NCBInr) database (release date: November 29, 2013), species restriction to *R. communis* and Viridiplantae (green plants) only when no proteins matched in *R. communis* using the MASCOT interface (Version 2.5, Matrix Science, London, UK). The following search parameters were used: no molecular weight restriction, one allowed missed trypsin cleavage, fixed modification of carbamidomethyl (C), variable modification of oxidation (Met), the peptide tolerance of 100 ppm, fragment mass tolerance of ± 0.4 Da and peptide charge of 1+. Protein identifications were validated with at least 3 peptides matched, keratin contamination was removed, and the MOWSE threshold was set over 60 (*P* < 0.05). The highest scoring peptide was then blast searched against the NCBI database and the protein was identified according to the alignment.

The functions of the identified proteins were searched against the UniProt (http://www.uniprot.org) and NCBI protein (http://www.ncbi.nlm.nih.gov) databases as described by Chen et al. (2014).

### Western blot analysis

Western blot analysis was performed as described previously (Hu et al., 2016). The protein samples were separated by 12% w/v standard SDS-PAGE and then electroblotted onto polyvinylidene difluoride membranes. After transfer, the membranes were probed with the appropriate primary antibodies and anti-rabbit IgG horseradish peroxidase conjugated to alkaline phosphatase (Abcam, UK, 1:5000 dilution) to detect the primary antibodies. The primary antibodies for the RuBisCO large subunit (RuBisCO LSU; Agrisera, Sweden), ATP synthase (ATPase; Agrisera) and chloroplast Cu/Zn superoxide dismutase (Cu/Zn SOD; Agrisera) were diluted 1:5000, 1:2000, and 1:1000, respectively. β-actin (1:5000; Santa Cruz, CA, USA) was used as an internal control. The signals were detected using an enhanced chemiluminescence kit (TIAN-GEN, China) according to the manufacturer's instructions.

### Statistical analysis

Values in figures are expressed as means ± SE. The statistical significance of the data was analyzed using univariate analysis of variance (*P* < 0.05) (One-way ANOVA; SPSS for Windows, version 16.0).

## Results

### Yield parameters in two castor varieties

By reasonably increasing the planting densities of Zhebi 100 (12,000 seedlings/hm^2^) and Zhebi 26 (36,000 seedlings/hm^2^), we demonstrated that Zhebi 26 could achieve a higher yield than Zhebi 100 (Table 1).

**Table 1:** **Yield parameters of Zhebi 100 and Zhebi 26 seedlings in different planting density**.

**Variety**	**Planting density (seedling/hm^2^)**	**Seed yield (g/seedling)**	**Seed yield (kg/hm^2^)**
Zhebi 100	8000	401.5 ± 8.5 a	3212 ± 81.3 b
	12,000	318.2 ± 6.2 b	3818.4 ± 94.2 a
	24,000	127.5 ± 3.5 c	3060 ± 76.1 c
	36,000	67.9 ± 3.1 d	2444.4 ± 66.7 d
	48,000	42.2 ± 2.8 e	2025.6 ± 47.9 e
Zhebi 26	8000	171.2 ± 4.3 a	1369.6 ± 35.2 d
	12,000	166.9 ± 4.9 a	2002.8 ± 51.5 c
	24,000	157.6 ± 4.1 b	3782.4 ± 61.2 b
	36,000	125.4 ± 3.6 c	4514.4 ± 72.4 a
	48,000	78.4 ± 2.7 d	3763.2 ± 59.6 b

*Data are means ± SE of eight replicates for seed yield (g/seedling) or four replicates for seed yield (kg/hm^2^). Means with different letters in the same column are significantly different (P < 0.05) with regard to different planting density in each castor varieties*.

### Morphological and physiological differences between *ricinus communis* varieties

**Figure 1:**
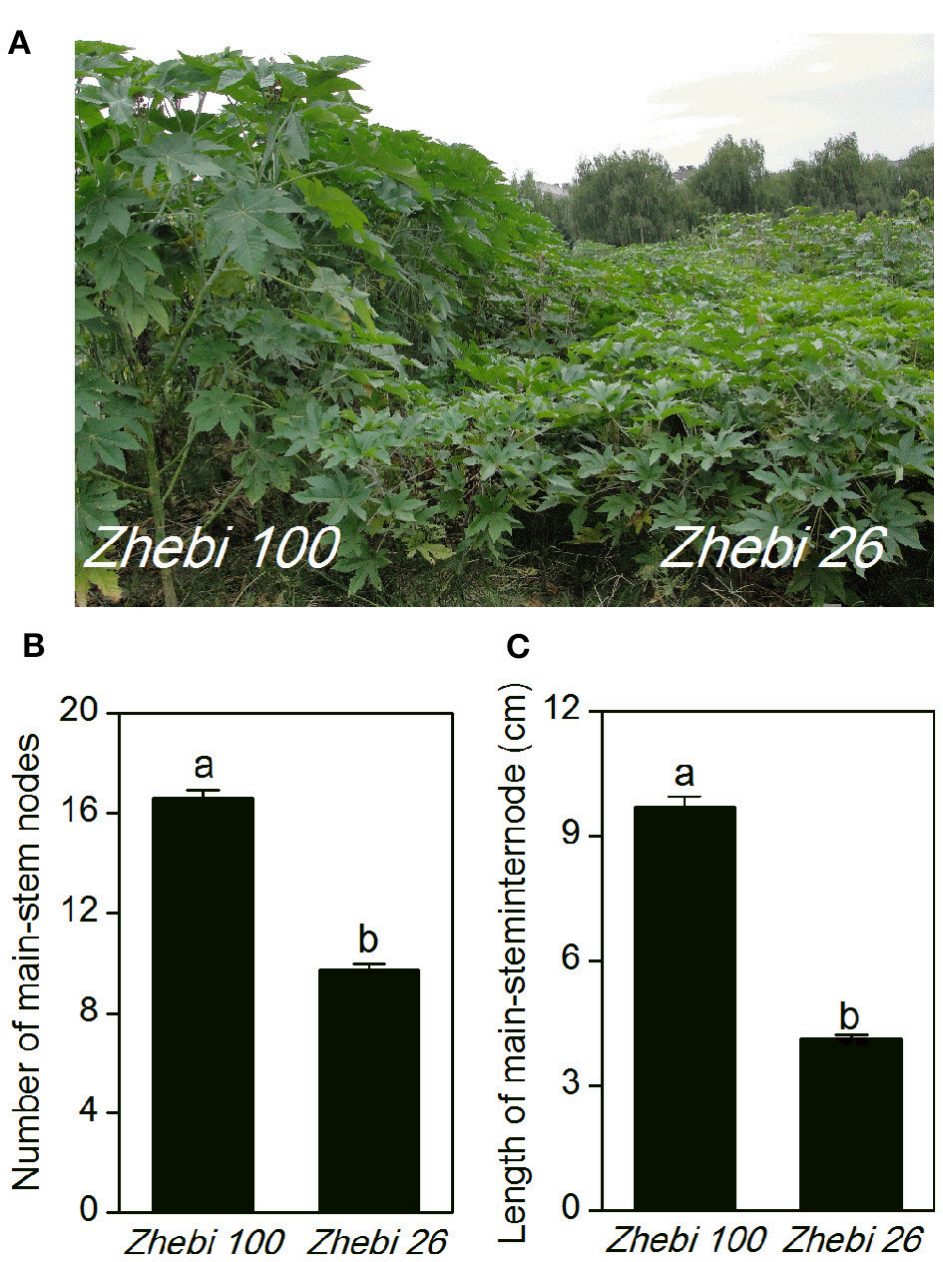
**Morphological parameters in two castor varieties. (A)** Phenotypes of the Zhebi 100 (left) and Zhebi 26 (right). **(B)** Number of main-stem nodes. **(C)** Length per node of main stem. Different letters above columns indicate significant difference at *P* < 0.05.

In the field experiment, we observed that the number of nodes and length per node of the main stem were higher in Zhebi 100 than in Zhebi 26 (Figure 1). Other parameters were also higher in Zhebi 100, including main stem length, main stem internode length, main stem diameter, central cavity, number of total branches, number of effective branches, and leaf thickness (Table 2).

**Table 2:** **Selected branch parameters of Zhebi 100 and Zhebi 26 seedlings**.

**Parameter**	**Zhebi 100**	**Zhebi 26**
Main-stem length (cm)	162.18 ± 2.06^*^	40.38 ± 1.24
Length of first branch (cm)	68.6 ± 3.06^*^	25.1 ± 2.11
Main stem diameter (cm)	3.86 ± 0.05^*^	2.43 ± 0.04
Central cavity (mm)	3.01 ± 0.03^*^	1.47 ± 0.02
Number of total branches	6.01 ± 0.09^*^	3.01 ± 0.05
Number of effective branches	2.70 ± 0.02^*^	2.00 ± 0.02
Leaf thickness (mm)	0.79 ± 0.02	0.83 ± 0.02

*Data are means ± SE of ten replicates. Means with asterisks in the same row are significantly different (P < 0.05) with regard to the same parameter comparing Zherbi 100 with Zherbi 26*.

After 20–125 growth days, the dry weights of different organs during different developmental stages were measured in the two varieties (Table 3). The roots and aerial parts of Zhebi 100 showed higher biomass accumulation. Notably, compared with Zhebi 100, the flowering and maturing times of Zhebi 26 were earlier.

**Table 3:** **Biomass of various plant organs between Zhebi 100 and Zhebi 26 seedlings**.

**Growth days**	**Species**	**Dry weight (g seedling^−1^)**				
		**Root**	**Stem**	**Leave**	**Flower**	**Seed**
20	*Zhebi 100*	0.15 ± 0.01	0.71 ± 0.06	1.26 ± 0.06		
	*Zhebi 26*	0.14 ± 0.01	0.73 ± 0.04	1.35 ± 0.02		
30	*Zhebi 100*	1.81 ± 0.07	5.79 ± 0.10	14.87 ± 0.52		
	*Zhebi 26*	1.86 ± 0.06	7.05 ± 0.04^*^	17.63 ± 0.43 ^*^	0.57 ± 0.04	
45	*Zhebi 100*	7.47 ± 0.18 ^*^	19.43 ± 0.31^*^	23.67 ± 0.21	0.62 ± 0.02	0.09 ± 0.01
	*Zhebi 26*	6.66 ± 0.09	18.17 ± 0.70	23.33 ± 0.23	3.81 ± 0.18^*^	1.10 ± 0.06^*^
65	*Zhebi 100*	25.47 ± 0.90 ^*^	110.27 ± 3.98^*^	86.70 ± 1.86^*^	37.00 ± 1.31	24.60 ± 2.02
	*Zhebi 26*	18.67 ± 1.03	62.13 ± 4.15	47.03 ± 1.09	71.10 ± 1.78^*^	40.80 ± 1.35^*^
85	*Zhebi 100*	45.67 ± 0.80	185.97 ± 4.95^*^	97.27 ± 1.23	103.47 ± 2.65	89.53 ± 4.97
	*Zhebi 26*	46.17 ± 1.51	136.63 ± 2.64	134.97 ± 2.69^*^	146.20 ± 3.17^*^	125.30 ± 1.70^*^
105	*Zhebi 100*	85.30 ± 2.34 ^*^	306.03 ± 3.47^*^	193.53 ± 2.07^*^	171.07 ± 2.98^*^	152.70 ± 2.74^*^
	*Zhebi 26*	37.57 ± 0.84	102.07 ± 3.33	85.33 ± 2.17	139.20 ± 3.15	123.83 ± 1.61
125	*Zhebi 100*	54.10 ± 1.39 ^*^	294.57 ± 2.59^*^	94.80 ± 1.91^*^	175.53 ± 2.06^*^	155.23 ± 3.43^*^
	*Zhebi 26*	19.83 ± 1.50	88.90 ± 1.06	39.07 ± 1.39	107.10 ± 3.28	

*Data are means ± SE of four replicates. Means with asterisks in the same column are significantly different (P < 0.05) with regard to the same growth day under 12,000 seedlings/hm^2^ in Zhebi 100, 36,000 seedlings/hm^2^ in Zhebi 26, respectively*.

However, there was no significant difference in non-photochemical quenching (NPQ) or chlorophyll *a* between Zhebi 100 and Zhebi 26 (Table 4). But, almost all other physiological and photosynthetic parameters were obviously higher in Zhebi 100, including Pn, stomatal condunctance (Gs), intercellular CO_2_ (Ci), transpiration rate (E), minimal fluorescence (F0), variabl efluorescence (Fv), photochemical quenching coefficient (qP), efficiency of open reaction centers (Fv/Fm), electronic transport ratio (ETR), and chlorophyll *b*. Yet, compared with Zhebi 100, increased planting density contributed to higher LAI in Zhebi 26.

**Table 4:** **Selected photosynthetic parameters of Zhebi 100 and Zhebi 26 seedlings**.

**Parameter**	**Zhebi 100**	**Zhebi 26**
Leaf area index (LAI, m^2^ m^−2^)	1.23 ± 0.02	1.53 ± 0.03^*^
Net photosynthetic rate (Pn, umol m^−2^ s^−1^)	36.42 ± 0.79^*^	27.94 ± 0.45
Stomatal condunctance (Gs, mmol m^−2^ s^−1^)	1.57 ± 0.03^*^	1.33 ± 0.04
Intercellular CO_2_ (Ci, umol m^−2^ s^−1^)	303.76 ± 5.33^*^	255.53 ± 2.27
Transpiration rate (E, mmol m^−2^ s^−1^)	7.07 ± 0.09 ^*^	6.87 ± 0.08
Minimal fluorescence (F0,umol m^−2^ s^−1^)	373.19 ± 4.16^*^	365.67 ± 2.60
Variabl efluorescence (F*v*, umol m^−2^ s^−1^)	1514.43 ± 16.39^*^	1301.67 ± 16.78
Efficiency of open reaction centers (Fv/Fm, umol m^−2^ s^−1^)	0.90 ± 0.01^*^	0.65 ± 0.02
Photochemical quenching coefficient (qP)	0.34 ± 0.005^*^	0.30 ± 0.002
Non-photochemical quenching (NPQ, umol m^−2^ s^−1^)	1.48 ± 0.02	1.46 ± 0.05
Electronic transport ratio (ETR, umol m^−2^ s^−1^)	61.60 ± 0.83^*^	50.33 ± 0.53
Chlorophyll a (mg g^−1^ DW)	2.13 ± 0.03	2.11 ± 0.01
Chlorophyll b (mg g^−1^ DW)	0.74 ± 0.01^*^	0.62 ± 0.02
Carotenoid pigment (mg g^−1^ DW)	0.27 ± 0.006	0.33 ± 0.005^*^

*Data are means ± SE of eight replicates. Means with asterisks in the same row are significantly different (P < 0.05) with regard to the same parameter under 12,000 seedlings/hm^2^ in Zhebi 100, 36,000 seedlings/hm^2^ in Zhebi 26, respectively*.

### Identification and functional classification of proteins changed in abundance comparing zherbi 100 and zherbi 26

**Figure 2:**
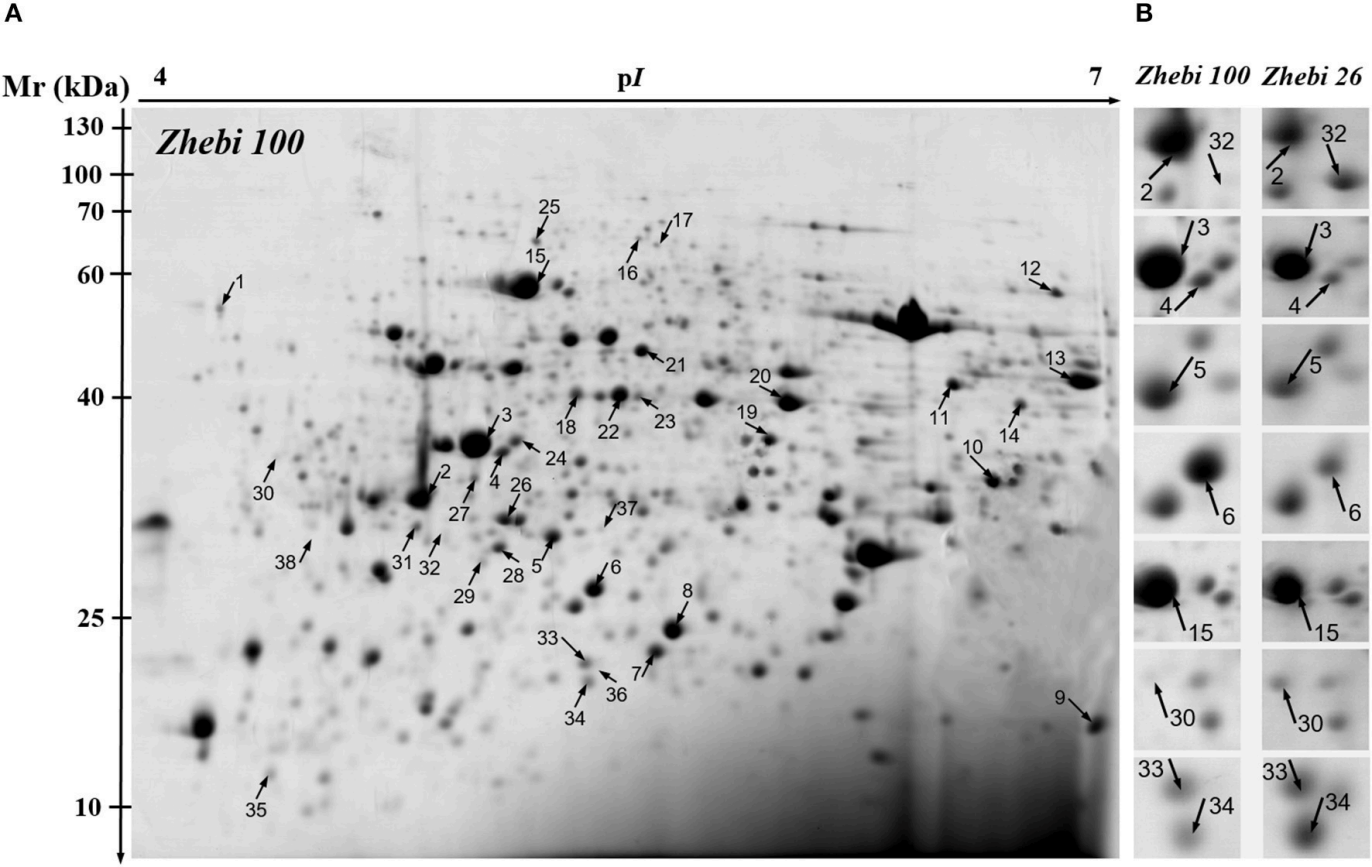
**2-DE analysis of proteins extracted from ***Ricinus communis*** leaves**. The numbers assigned to the protein spots correspond to those listed in Table 5. **(A)** Representative CBB R250-stained 2D gel of total proteins from Zhebi 100. Arrows indicate 38 spots showing at least 2-fold changes (*P* < 0.05) analyzed by MALDI-TOF/TOF MS. **(B)** The enlarged window shows some differential protein spots changed in abundance between Zhebi 100 and Zhebi 26.

**Figure 3:**
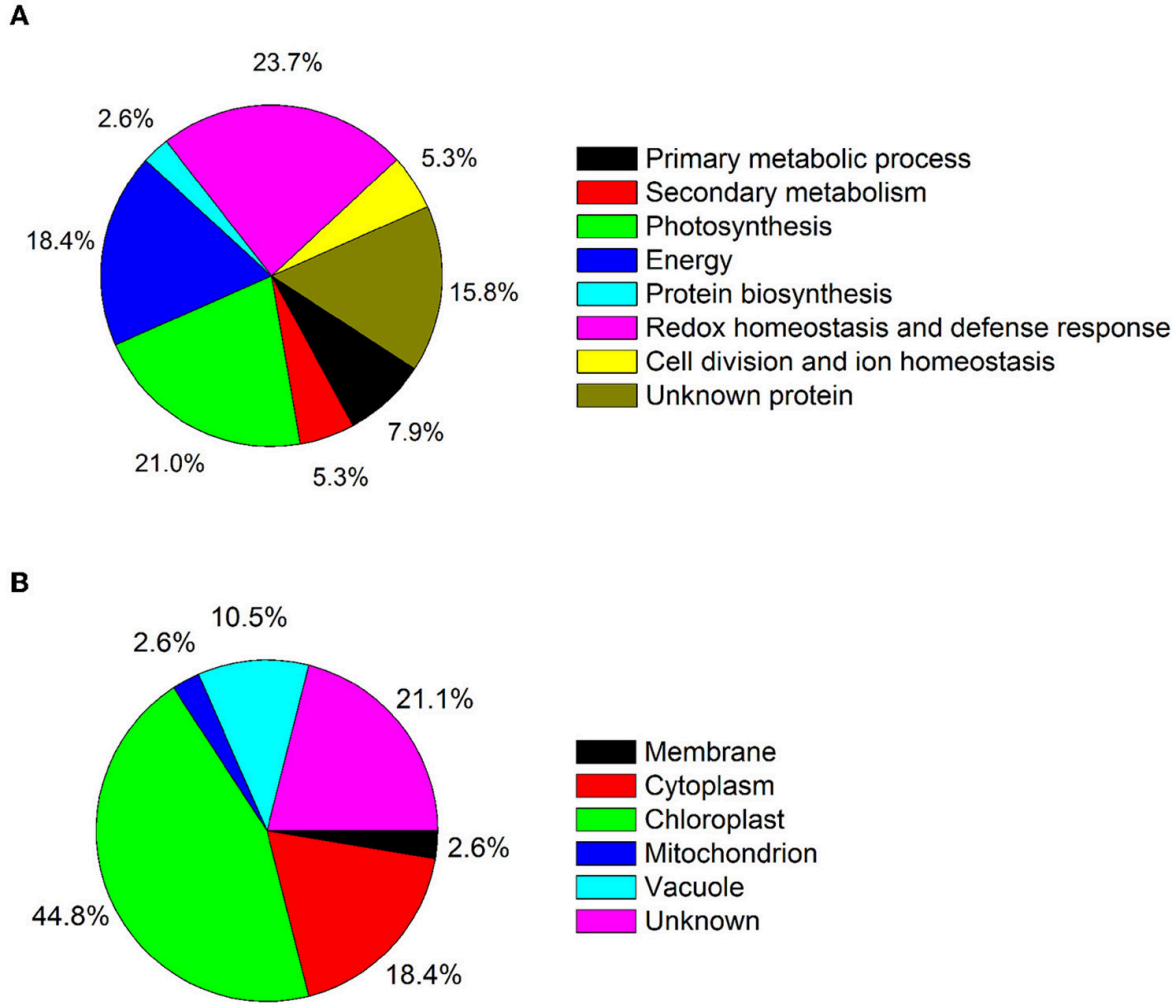
**(A)** Functional category distribution of 38 identified proteins. **(B)** Subcellular locations of the identified proteins.

Because leaves are the major photosynthetic organs of broad-leaved crops, and leaf formation is a basic aspect of plant development and crop productivity. So we used leaf samples for experimental material. To better understand the differences in plant productivity between a high-stalk variety and dwarf variety, we carried out a comparative proteomic study comparing Zhebi 100 with Zhebi 26. A total of 38 protein spots were identified that were significantly different in abundances in the two-dimensional electrophoresis analysis of the two varieties (Figure 2, Table 5). These differential proteins belonged to a wide range of metabolic pathways. The data indicated that the identified proteins falling into eight functional categories according to their biological functions (Figure 3A), with the main groups being redox homeostasis and defense responses (23.7%), photosynthesis (21.0%), energy (18.4%), primary metabolic processes (7.9%), secondary metabolism, cell division and ion homeostasis (5.3%), and protein biosynthesis (2.6%).

**Table 5:** **Proteins identities using MALDI-TOF/TOF MS in Zherbi 100 compared with Zherbi 26 at the highest yield planting density**.

**Spot^a^**	**NCBI accession^b^**	**Protein identity^c^**	**Thero. kDa/p*I*^d^**	**Exper. kDa/p*I*^e^**	**Pep. Count^f^**	**Protein score^g^**	**C^h^**	**Species**
**PRIMARY METABOLIC PROCESS**								
13	gi|255543455	Glyceraldehyde 3-phosphate dehydrogenase	43.37/8.14	42.29/6.75	21	741	I	*Ricinus communis*
4	gi|255540407	2-deoxyglucose-6-phosphate phosphatase	35.03/7.92	35.70/5.65	18	454	I	*Ricinus communis*
11	gi|255559448	NAD dependent epimerase/dehydratase	43.23/8.75	41.90/6.50	15	580	I	*Ricinus communis*
**SECONDARY METABOLISM**								
29	gi|255565419	Lactoylglutathione lyase	26.41/9.11	28.21/5.61	10	197	I	*Ricinus communis*
36	gi|223527364	Cyanate hydratase	18.62/5.68	19.44/5.83	8	82	D	*Ricinus communis*
**PHOTOSYNTHESIS**								
2	gi|255557387	Chlorophyll A/B binding protein	28.30/5.29	32.49/5.37	6	280	I	*Ricinus communis*
3	gi|255562761	oxygen-evolving enhancer protein 1	35.45/5.58	36.25/5.60	17	839	I	*Ricinus communis*
5	gi|255567170	Chlorophyll A/B binding protein	29.36/6.85	29.76/5.74	6	79	I	*Ricinus communis*
6	gi|663085383	Ribulose-1,5-bisphosphate carboxylase/oxygenase large subunit	23.26/6.93	26.61/5.82	10	329	I	*Euphorbia mellifera* var.*canariensis*
9	gi|255582745	Ribulose bisphosphate carboxylase small chain	21.07/9.03	15.13/6.77	14	484	I	*Ricinus communis*
10	gi|126166001	Ribulose-1,5-bisphosphate carboxylase/oxygenase large subunit	51.90/6.30	33.47/6.58	25	488	I	*Astraea lobata*
18	gi|255559812	Photosystem II stability/assembly factor HCF136	43.41/7.11	40.63/5.79	14	732	I	*Ricinus communis*
24	gi|255562761	Oxygen-evolving enhancer protein 1	35.45/5.58	36.58/5.67	16	537	I	*Ricinus communis*
**ENERGY**								
14	gi|255585546	Malate dehydrogenase	35.98/6.40	39.53/6.63	16	730	I	*Ricinus communis*
15	gi|339516172	ATP synthase CF1 beta subunit	53.71/5.11	56.94/5.69	20	626	I	*Ricinus communis*
16	gi|255544516	ATP synthase alpha subunit vacuolar	63.49/5.31	65.05/5.91	39	1370	I	*Ricinus communis*
19	gi|255586297	Ferredoxin-NADP reductase	38.65/9.00	36.53/6.15	16	512	I	*Ricinus communis*
20	gi|255543861	Fructose-bisphosphate aldolase	43.14/7.55	39.73/6.19	14	495	I	*Ricinus communis*
22	gi|255554879	ATP synthase gamma chain 2	41.54/5.65	40.55/5.87	18	577	I	*Ricinus communis*
23	gi|255581400	Fructose-bisphosphate aldolase	42.95/6.78	40.40/5.91	14	553	I	*Ricinus communis*
**PROTEIN BIOSYNTHESIS**								
21	gi|255540493	Elongation factor tu	50.26/5.99	46.58/5.91	17	712	I	*Ricinus communis*
**REDOX HOMEOSTASIS AND DEFENSE RESPONSE**								
1	gi|255589194	Peroxidase 22 precursor	21.12/4.73	53.25/4.61	4	291	I	*Ricinus communis*
7	gi|255565475	superoxide dismutase (Cu-Zn)	21.67/6.28	21.32/5.94	5	301	I	*Ricinus communis*
8	gi|255544369	Cytochrome b6-f complex iron-sulfur subunit	23.79 /8.22	23.65/5.97	7	256	I	*Ricinus communis*
12	gi|255556504	Dihydrolipoamide dehydrogenase	54.14/6.96	55.95/6.69	30	1040	I	*Ricinus communis*
17	gi|255540797	Betaine-aldehyde dehydrogenase	55.69/5.48	64.01/5.94	14	475	I	*Ricinus communis*
28	gi|255549438	Glutathione s-transferase	24.53/5.16	29.08/5.64	13	356	I	*Ricinus communis*
30	gi|255539971	superoxide dismutase (fe)	34.71/4.86	35.35/4.83	8	180	D	*Ricinus communis*
33	gi|255581166	Major latex protein	17.36/5.42	20.20/5.80	17	421	D	*Ricinus communis*
34	gi|255587426	Major latex protein	16.85/5.43	18.50/5.81	11	266	D	*Ricinus communis*
**CELL DIVISION AND ION HOMEOSTASIS**								
25	gi|255558698	Cell division protein ftsH	75.50/6.43	64.57/5.71	36	1250	I	*Ricinus communis*
31	gi|255571441	Ferritin	28.57/5.25	30.38/5.36	16	461	D	*Ricinus communis*
**UNKNOWN PROTEIN**								
35	gi|255548059	Hypothetical protein RCOM_1340080	15.29/8.95	11.95/4.80	3	90	D	*Ricinus communis*
37	gi|255552269	Stem-specific protein TSJT1	25.50/5.56	30.33/5.83	11	463	D	*Ricinus communis*
38	gi|508716126	f-box and leucine rich repeat domains containing protein	166.40/5.20	29.54/4.95	34	68	D	*Theobroma cacao*
26	gi|255582834	Ricin-agglutinin family protein	33.79/5.16	30.90/5.65	13	557	I	*Ricinus communis*
27	gi|255550621	ricin-agglutinin family protein	34.69/5.99	33.78/5.59	15	913	I	*Ricinus communis*
32	gi|255582834	Ricin-agglutinin family protein	33.79/5.16	30.53/5.46	11	731	D	*Ricinus communis*

a *Spot. is spot number of the unique differential proteins changed in abundance*.

b *Database accession numbers according to NCBInr*.

c *The name of the proteins identified by MALDI-TOF/TOF MS*.

d *Theoretical mass (kDa) and pI of identified proteins*.

e *Experimental mass (kDa) and pI of identified proteins*.

f *Number of the matched peptides*.

g *The Mascot searched score against the database NCBInr*.

h *Spot abundance change. D stands for decreased abundance of proteins, I stands for increased abundance of protein*.

In subcellular localization analysis, the majority of these proteins were located in the chloroplast (44.8%), followed by in the cytoplasm (18.4%), vacuole (10.5%), membrane (2.6%), and mitochondrion (2.6%) (Figure 3B).

### Protein abundance analysis by western blot

**Figure 4:**
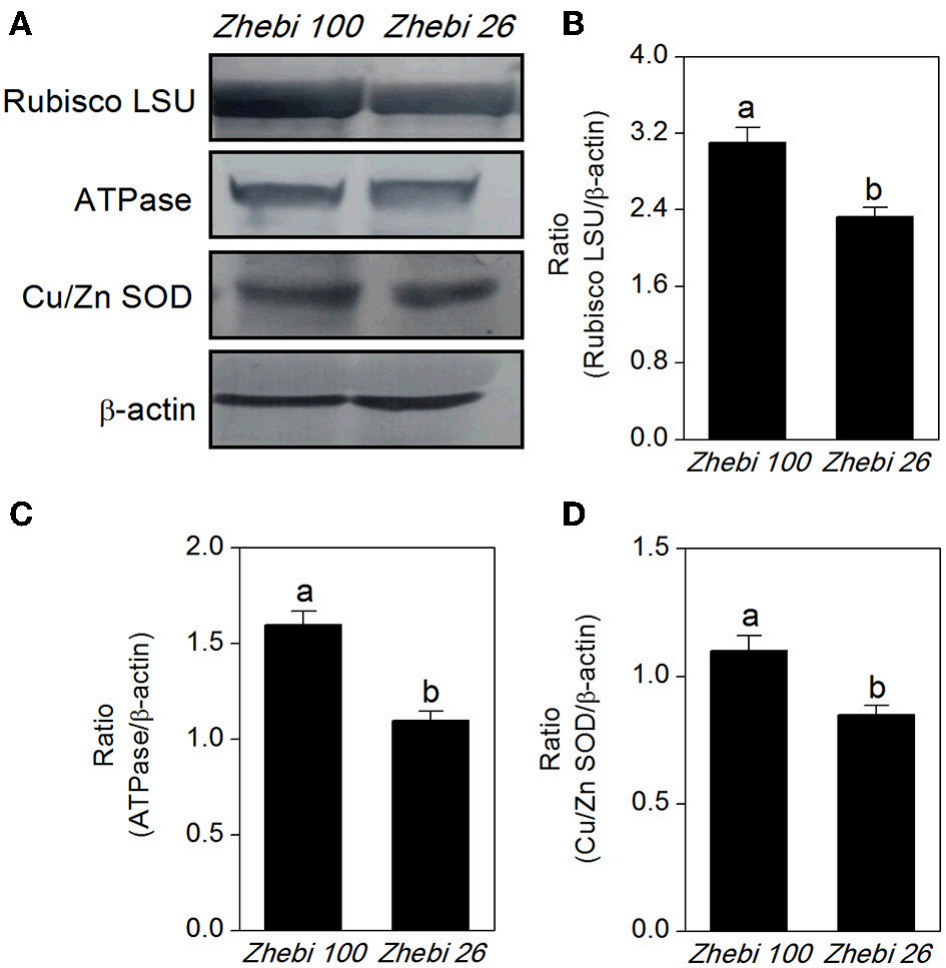
**(A)** The protein abundance levels of the ribulose-1,5-bisphosphate carboxylase large subunit (RuBisCO LSU), ascorbate peroxidase (APX) and superoxide dismutase (Cu-Zn) (Cu/Zn SOD) in Zhebi 100 and Zhebi 26 from western blotting. The relative abundance levels of RuBisCO LSU **(B)**, ATPase **(C)**, Cu/Zn SOD **(D)** were analyzed with the Quantity One software. β-actin was used as the internal control. Bars with different letters are significantly different from each other (*P* < 0.05).

The proteomic results revealed that the abundances of RuBisCO (spots 6, 10), ATP synthase (spots 15, 16, 22), and superoxide dismutase (Cu-Zn) (spot 7) were decreased in Zhebi 26 (Table 5). As shown in Figure 4, the protein abundance levels of RuBisCO LSU, ATPase and Cu/Zn SOD were indeed significantly decreased in Zhebi 26 according to Western blot analysis.

### Quality assessment of final laboratory-refined oil in the two castor varieties

In comparing with Zhebi 26, there does not appear to have major differences in total oil content in Zhebi 100 (Table 6). However, Zhebi 100 contains a significantly higher content of palm acid, whereas Zhebi 26 contains significantly higher amounts of ricinoleic acid, oleic acid, and linoleic acid.

**Table 6:** **Quality assessment of final laboratory-pressed oil between Zhebi 100 and Zhebi 26**.

**Species**	**Oil content (%)**	**Ricinoleic acid (%)**	**Palmitic acid (%)**	**Oleic acid (%)**	**Linoleic acid (%)**
Zhebi 100	54.13 ± 0.66	84.13 ± 0.66	1.55 ± 0.02^*^	4.34 ± 0.03	5.10 ± 0.03
Zhebi 26	54.13 ± 0.50	86.10 ± 0.51^*^	1.27 ± 0.04	4.68 ± 0.02^*^	5.83 ± 0.07^*^

*Data are means ± SE of four replicates. Means with asterisks in the same column are significantly different (P < 0.05) under 12,000 seedlings/hm^2^ in Zhebi 100, 36,000 seedlings/hm^2^ in Zhebi 26, respectively*.

## Discussion

### Metabolism related proteins

Leaf formation is a basic aspect of plant development and crop productivity because leaves are the major photosynthetic organs of broad-leaved crops. An understanding of the processes underlying the differences between *R. communis* varieties thus provides an insight into a basic biological process, modulation of which may have far-reaching significance for strategies to improve crops yield and the environment. Consistent with the growth and development indexes, Zhebi 100 showed higher biomass accumulation in its aerial parts and roots during different developmental stages (Table 3). Numerous early studies reported that glyceraldehyde 3-phosphate dehydrogenase is a key enzyme involved in glycolysis and cellular energy production associated with plant development (Giegé et al., 2003; Hu et al., 2014a). In this research, we found that glyceraldehyde-3-phosphate dehydrogenase C2 (spot 13) is increased expected in Zhebi 100.

A previous study reported that 2-deoxyglucose-6-phosphate phosphatase (spot 4) displays sugar-phosphatase activity, and plays an important role in plant carbon metabolism (Baskin et al., 2001). Furthermore, NAD dependent epimerase/dehydratase (spot 11) has racemase and epimerase activities, acting on carbohydrates and their derivatives, and takes part in pectin-related carbohydrate metabolic processes (Wang et al., 2008). In this study, the majority of protein spots (spots 4, 11) related to primary carbohydrate metabolism increased in abundance in Zhebi 100. This indicates that carbon metabolism is more active in Zhebi 100, providing more resources for tissue formation during development.

### Photosynthesis, energy pathway and protein biosynthesis related proteins

Zhebi 100 leaves showed higher abundance of photosynthesis and energy pathway related proteins than Zhebi 26 (Table 5). This was supported by the observations that Zhebi 100 leaves have a higher total chlorophyll (a + b) content and net photosynthesis rate (Table 4). These results suggest that proteins involved in carbon assimilation, folding and assembly, and energy metabolism may work synchronously and show a correlation to the increased photosynthetic capacity in Zhebi 100 leaves. The key enzymes involved in these above processes are assembled into the RuBisCO complex and its activator RuBisCO activase (Portis and Parry, 2007); these enzymes showed higher accumulation levels in Zhebi 100 (spots 6, 9, 10; Table 5). Additionally, Western blot analysis showed that the RuBisCO large subunit was increased in abundance in Zhebi 100 leaves (Figure 4), Western blot result is consistent with the proteomic data for the selected protein at the highest yield planting density. These results support our speculation that a lower photosynthetic rate and primary carbon metabolism might be the reason for the slower plant growth and development in Zhebi 26. This was supported by Pn and chlorophyll fluorescence measurements; these photosynthetic indexes were markedly lower in Zhebi 26 as shown in Table 4. Moreover, similar patterns of decreased biomass accumulation of aerial parts were observed in Zhebi 26 compared with Zhebi 100 at the 85th day after planting (Table 3). Because the mature dates are different between Zhebi 26 and Zhebi 100: Zhebi 26's mature date is around the 85th day after planting (the growth of the aerial parts of Zhebi 26 stopped after the mature date), whereas Zhebi 100 continues to grow until its mature date around the 105th day. Elongation factor Tu (spot 21, Table 5), an essential component for protein synthesis (Hu et al., 2014c), also showed higher abundance in Zhebi 100 leaves. We speculate that Zhebi 100 has higher biosynthetic activity, and the participation of elongation factor Tu is required for the correct folding of the newly synthesized proteins.

In relation to energy production, malate dehydrogenase (spot 14, Table 5), which is implicated in NADPH photoactivation (Valledor et al., 2010), increased in abundance in Zhebi 100 leaves. Moreover, previous studies have indicated that sufficient ATP is necessary for plant growth and development (Jiang et al., 2007; Hu et al., 2014b). ATP is mainly produced by carbohydrate metabolism, such as glycolysis and the tricarboxylic acid cycle. Numerous papers have reported that several enzymes involved in glycolysis and the tricarboxylic acid cycle are associated with plant development (Palma et al., 2011; Cui et al., 2012). In this study, we identified seven proteins related to energy production pathways (Table 5). Among them, fructose-bisphosphate aldolase (spots 20, 23), an essential enzyme involved in the glycolytic pathway, showed enhanced abundance in Zhebi 100. We also found that the ATP synthase CF1 beta subunit (spot 15), ATP synthase vacuolar alpha subunit (spot 16) and ATP synthase gamma chain 2 (spot 22) had increased abundance in Zhebi 100. Ferredoxin-NADP reductase is a ubiquitous flavoenzyme that delivers NADPH or low potential one-electron donors (ferredoxin) to redox-based metabolisms in plastids and mitochondria (Ceccarelli et al., 2004). Donation of electrons by ferredoxin has been demonstrated in many other plastid enzymes, which may be essential for sufficient energy production in Zhebi 100.

**Figure 5:**
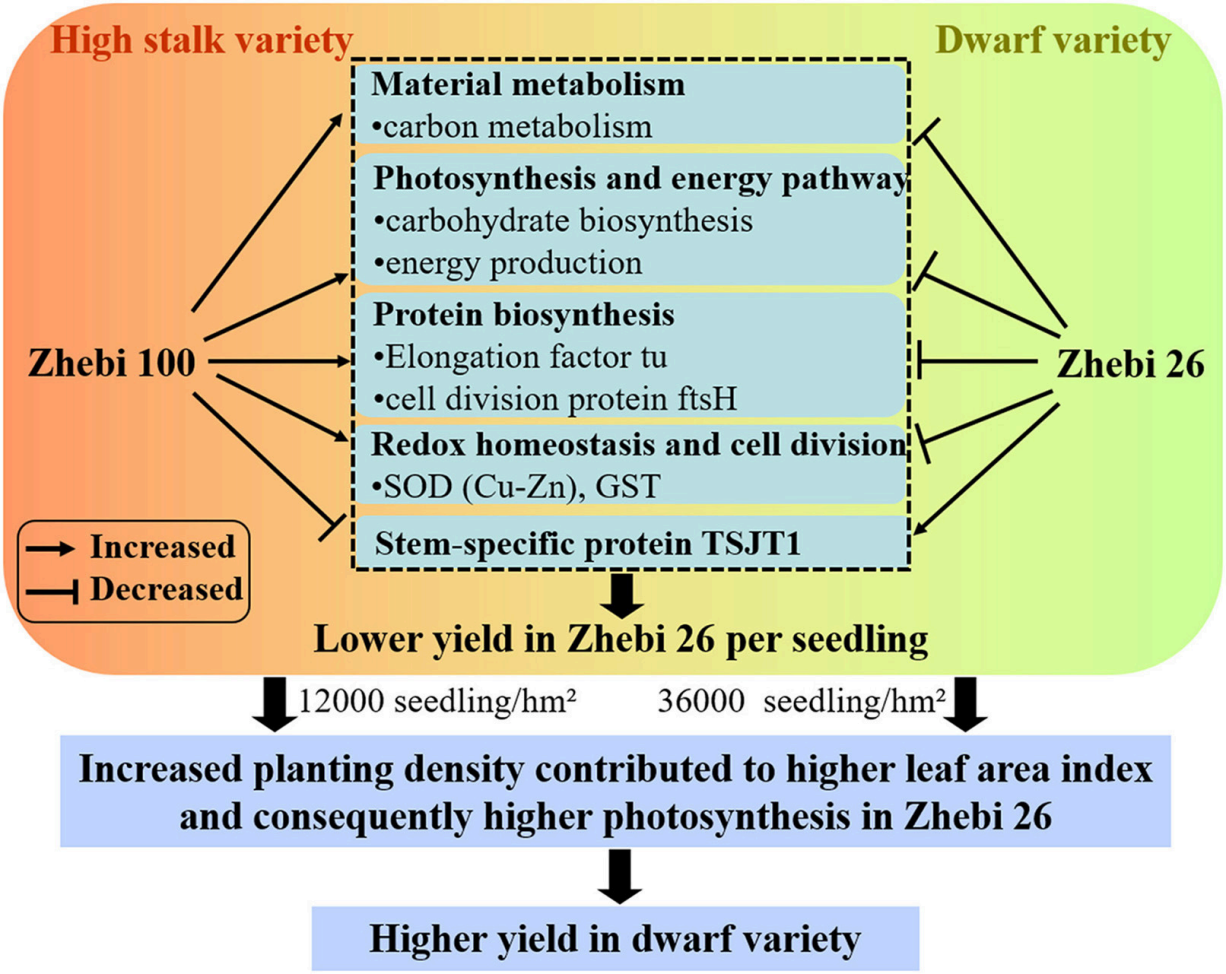
**A schematic representation of different growth and development mechanisms in Zhebi 100 and Zhebi 26 via regulation of diverse biological processes**.

Compared with Zhebi 26, Zhebi 100 displays a higher number of proteins increased in abundance (Table 5). These proteins are mainly related to chloroplast electron transfer chain, carbohydrate biosynthesis, and energy production, which may be the reason that Zhebi 100 possesses the higher photosynthesis and energy metabolic activity than Zhebi 26. In addition, we demonstrated that Zhebi 26 (36,000 seedlings/hm^2^) can achieve a higher yield than Zhebi 100 (12,000 seedlings/hm^2^) through increased planting density (Table 1). The reasonable explanation is that increased planting density contributes to higher LAI in Zhebi 26 compared with Zhebi 100 (Table 4). Higher LAI means higher light interception by plants, higher Pn, and consequently higher productivity. Therefore, Zhebi 26 can achieve a higher yield than Zhebi 100 through reasonably close planting (Figure 5).

### Redox homeostasis and defense response related proteins

Growing evidence indicates that, in redox homeostasis, reactive oxygen species (ROS) play a dual role in plant biology as both toxic byproducts of aerobic metabolism and key regulators of growth, development and defense pathways (Mittler et al., 2004; Yan et al., 2006). As the most important reaction in the cell elongation process, loosening of plant cell walls is associated with the production of ROS (Foreman et al., 2003; Kwon et al., 2007; Yang et al., 2008), which could damage plant cell if not removed efficiently, therefore increased antioxidation capacity is the hallmark of fast plant growth (Cui et al., 2012). However, plants can regulate ROS levels through complex mechanisms such as ROS scavenging with superoxide dismutase (Goossens et al., 2003) and glutathione S-transferase (GST) (Apel and Hirt, 2004; Hu et al., 2014b). In Zhebi 100, many antioxidant enzymes associated with redox homeostasis and antioxidation response were increased in abundance, including a peroxidase 22 precursor (spot 1), SOD (Cu-Zn) (spot 7), a cytochrome b6-f complex iron-sulfur subunit (spot 8), dihydrolipoamide dehydrogenase (spot 12), betaine-aldehyde dehydrogenase (spot 17) and GST (spot 28). Furthermore, Western blot analysis showed that the protein abundance of SOD (Cu-Zn) was increased in Zhebi 100 leaves (Figure 4), which was consistent with the proteomic data at the highest yield planting density. The findings presented above, together with previously published data, strongly suggest that ROS-mediated cell expansion may be an important mechanism regulating castor bean growth and development. On the other hand, enhanced abundance of these proteins may also imply that the anti-oxidative defense system and resistance to environmental stress is increased in Zhebi 100.

### Proteins related to other functions

Plant internode elongation is correlated with cell division and elongation (Cui et al., 2012). The increased abundance of the cell division protein ftsH (spot 25) is potentially important for the longer internodes in Zhebi 100 compared with Zhebi 26. In addition, little is known about the function of the stem-specific protein TSJT1 in plant growth and development. Notably, compared with Zhebi 26, the abundance of TSJT1 (spot 37) was decreased in Zhebi 100. As shown in Figure 1 and Table 2, Zhebi 26 exhibited a wide range of morphological phenotypes, such as dwarfism, compared with Zhebi 100. These results suggest that the intriguing stem-specific protein TSJT1 may act as a negative regulator in castor internode development. The specific function of this protein needs further study in the future.

In plants, ferritin is an essential regulator of iron homeostasis (Ravet et al., 2008). In our study, the abundance of ferritin (spot 31) was increased in Zhebi 26. Ferritin accumulation is induced by an excess of iron, as well as by abscisic acid, photoinhibition and ozone (Murgia et al., 2002). A secondary function of ferritin is protection from the oxidative stress of ROS (Ravet et al., 2008). In leaves, ferritin is an iron source at early stages of development for the synthesis of iron-containing proteins involved in photosynthesis (Briat and Lobréaux, 1997; Kobayashi and Nishizawa, 2012). The specific role of ferritin in redox homeostasis and photosynthesis response needs to further study in the future.

## Author contributions

WH and GS: Conceived and designed the research; WH, LC, XQ, HL, JW, and YB: Performed the research; NH, RH, and LS: Analyzed the data; WH and GS: Wrote the paper; and HZ: Revised this paper.

## Funding

This study was supported by the State Key Laboratory Breeding Base for Zhejiang Sustainable Pest and Disease Control (2010DS700124-KF1405 and KF1612, 2015-cxzt-01), the State Key Laboratory of Silkworm Genome Biology (20120007), the National Natural Science Foundation of China (31571718, 31402140) and of Zhejiang province (LY15C020002), the International Cooperation, Innovation Programs of Zhejiang Academy of Agricultural Sciences (2014CX005), and the Shaoxing 330 Overseas Elites Program to GS.

### Conflict of interest statement

The authors declare that the research was conducted in the absence of any commercial or financial relationships that could be construed as a potential conflict of interest.
