# Supplementary material for: Morphological, Physiological and Proteomic Analyses Provide Insights into the Improvement of Castor Bean Productivity of a Dwarf Variety in Comparing with a High-Stalk Variety
Source: Front Plant Sci. 2016 Sep 29;7:1473. doi: 10.3389/fpls.2016.01473 (PMC5040714; doi:10.3389/fpls.2016.01473)
Supplement: Supplementary file 1 [file Table1.DOCX]

Table S1. Lists of all identified peptides in differentially expressed proteins identified in *Ricinus communis*.

| **Spot^a^** | **NCBI  accession^b^** | **Protein identity^c^** | **Pep. Count^d^** | **Protein score^e^** | **Peptide sequence** |
| --- | --- | --- | --- | --- | --- |
| **1** | gi\|255589194 | peroxidase 22 precursor | 4 | 291 | YFSNLLSGK |
|  |  |  |  |  | AQCFTFSQR |
|  |  |  |  |  | SGGPSWTNQLGR |
|  |  |  |  |  | FLDVGLNDNVDLVALSGGHTFGR |
| **2** | gi\|255557387 | chlorophyll A/B binding protein | 6 | 280 | FGEAVWFK |
|  |  |  |  |  | ELEVIHCR |
|  |  |  |  |  | NRELEVIHCR |
|  |  |  |  |  | SVSSGSPWYGPDR |
|  |  |  |  |  | WAMLGALGCVFPELLAR |
|  |  |  |  |  | VAGGPLGEVTDPIYPGGSFDPLGLADDPEAFAELK |
| **3** | gi\|255562761 | oxygen-evolving enhancer protein 1 | 17 | 839 | GSSFLDPK |
|  |  |  |  |  | VPFLFTIK |
|  |  |  |  |  | LTFDEIQSK |
|  |  |  |  |  | NAPPEFQNTK |
|  |  |  |  |  | RLTFDEIQSK |
|  |  |  |  |  | FCLEPTSFTVK |
|  |  |  |  |  | KFCLEPTSFTVK |
|  |  |  |  |  | GGSTGYDNAVALPAGGR |
|  |  |  |  |  | GTGTANQCPTIDGGLDK |
|  |  |  |  |  | DGIDYAAVTVQLPGGER |
|  |  |  |  |  | GRGGSTGYDNAVALPAGGR |
|  |  |  |  |  | GTGTANQCPTIDGGLDKFSFK |
|  |  |  |  |  | LTYTLDEIEGPFEVGPDGTVK |
|  |  |  |  |  | FEEKDGIDYAAVTVQLPGGER |
|  |  |  |  |  | QLVATGKPESFSGEFLVPSYR |
|  |  |  |  |  | GGSTGYDNAVALPAGGRGDEEELAK |
|  |  |  |  |  | ITLSVTGSKPETGEIIGVFESVQPSDTDLGAK |
| **4** | gi\|255540407 | 2-deoxyglucose-6-phosphate phosphatase | 18 | 454 | NEEERK |
|  |  |  |  |  | LIDQALGK |
|  |  |  |  |  | MTAYFNK |
|  |  |  |  |  | EFIASLHK |
|  |  |  |  |  | APKNEEER |
|  |  |  |  |  | IFAGDVVPR |
|  |  |  |  |  | LLPLRPGVAK |
|  |  |  |  |  | KEFIASLHK |
|  |  |  |  |  | VAVCSTSNEK |
|  |  |  |  |  | EFIASLHKR |
|  |  |  |  |  | KLLPLRPGVAK |
|  |  |  |  |  | TELFMALIEK |
|  |  |  |  |  | IKIFAGDVVPR |
|  |  |  |  |  | ISFNDTFNER |
|  |  |  |  |  | TELFMALIEKK |
|  |  |  |  |  | AVSAIVSCLLGPER |
|  |  |  |  |  | ELGVTWDVDLYGELLK |
|  |  |  |  |  | KKPDPAIYTLAANTLAVDPSSCVVVEDSAIGLAAAK |
| **5** | gi\|255567170 | chlorophyll A/B binding protein | 6 | 79 | QLWFASK |
|  |  |  |  |  | QYFLGLEK |
|  |  |  |  |  | WLAYGEIINGR |
|  |  |  |  |  | FQDWAKPGSMGK |
|  |  |  |  |  | FAMLGAAGAIAPEILGK |
|  |  |  |  |  | FLGGSGDPAYPGGPLFNPLGFGK |
| **6** | gi\|663085383 | ribulose-1,5-bisphosphate carboxylase/oxygenase largesubunit | 10 | 329 | ALRLEDLR |
|  |  |  |  |  | DTDILAAFR |
|  |  |  |  |  | LTYYTPEYETK |
|  |  |  |  |  | LEDLRIPPAYTK |
|  |  |  |  |  | TFQGPPHGIQVER |
|  |  |  |  |  | DDENVNSQPFMR |
|  |  |  |  |  | DDENVNSQPFMRW |
|  |  |  |  |  | TFQGPPHGIQVERDK |
|  |  |  |  |  | LTYYTPEYETKDTDILAAFR |
|  |  |  |  |  | VTPQPGVPPEEAGAAVAAESSTGTWTTVWTDGLTSLDR |
| **7** | gi\|255565475 | superoxide dismutase (Cu-Zn) | 5 | 301 | GGHELSLTTGNAGGR |
|  |  |  |  |  | ALVVHELEDDLGK |
|  |  |  |  |  | GNSNVEGVVTLTQGDDGPTTVNVR |
|  |  |  |  |  | ALVVHELEDDLGKGGHELSLTTGNAGGR |
|  |  |  |  |  | HAGDLGNIVANADGVAEATIVDSQIPLSGPNAVIGR |
| **8** | gi\|255544369 | cytochrome b6-f complex iron-sulfur subunit | 7 | 256 | VPDMGKR |
|  |  |  |  |  | TGDAPWWA |
|  |  |  |  |  | GDPTYLVVEK |
|  |  |  |  |  | GDPTYLVVEKDR |
|  |  |  |  |  | VVFVPWVETDFR |
|  |  |  |  |  | GPAPLSLALAHADIDDGK |
|  |  |  |  |  | FICPCHGSQYNDQGR |
| **9** | gi\|255582745 | ribulose bisphosphate carboxylase small chain | 14 | 484 | ELDEAIK |
|  |  |  |  |  | SPGYYDGR |
|  |  |  |  |  | EVEYLLR |
|  |  |  |  |  | YWTMWK |
|  |  |  |  |  | IIGFDNVR |
|  |  |  |  |  | AYPNSFVR |
|  |  |  |  |  | EVEYLLRK |
|  |  |  |  |  | FETLSYLPDLTR |
|  |  |  |  |  | LPMFGCSDAVQVLK |
|  |  |  |  |  | KFETLSYLPDLTR |
|  |  |  |  |  | ELDEAIKAYPNSFVR |
|  |  |  |  |  | QVQCISFIAYKPPSFK |
|  |  |  |  |  | QVQCISFIAYKPPSFKDA |
|  |  |  |  |  | GWVPCLEFELEHGFVYR |
| **10** | gi\|126166001 | ribulose-1,5-bisphosphate carboxylase/oxygenase largesubunit | 25 | 488 | EGNEIIR |
|  |  |  |  |  | NHGMHFR |
|  |  |  |  |  | AVYECLR |
|  |  |  |  |  | NHGMHFR |
|  |  |  |  |  | AMHAVLDR |
|  |  |  |  |  | NEGRDLAR |
|  |  |  |  |  | AQAETGEIK |
|  |  |  |  |  | DTDILAAFR |
|  |  |  |  |  | DDFIEKDR |
|  |  |  |  |  | VALEACVQAR |
|  |  |  |  |  | QKNHGMHFR |
|  |  |  |  |  | EGNEIIRAASK |
|  |  |  |  |  | DNGLLLHIHR |
|  |  |  |  |  | FLFCAEAIYK |
|  |  |  |  |  | DITLGFVDLLR |
|  |  |  |  |  | LTYYTPDYQTK |
|  |  |  |  |  | DDENVNSQPFMR |
|  |  |  |  |  | MSGGDHIHAGTVVGK |
|  |  |  |  |  | DDENVNSQPFMR |
|  |  |  |  |  | EIKFEFQAMDTL |
|  |  |  |  |  | DRFLFCAEAIYK |
|  |  |  |  |  | WSPELAAACEVWK |
|  |  |  |  |  | GHYLNATAGTCEEMIK |
|  |  |  |  |  | LEGERDITLGFVDLLR |
|  |  |  |  |  | GHYLNATAGTCEEMIKR |
|  |  |  |  |  | DITLGFVDLLRDDFIEK |
|  |  |  |  |  | GGLDFTKDDENVNSQPFMR |
| **11** | gi\|255559448 | NAD dependent epimerase/dehydratase | 15 | 580 | FSEIVSAGGK |
|  |  |  |  |  | MKKPPFSR |
|  |  |  |  |  | TVWGDPAEVGK |
|  |  |  |  |  | NMHFYAEPR |
|  |  |  |  |  | DCEEWFFDR |
|  |  |  |  |  | RFMPSYGGCAR |
|  |  |  |  |  | DLDTVRPVADWAK |
|  |  |  |  |  | VVEGATFDVVLDNNGK |
|  |  |  |  |  | LCAQAAGLPVEIVHYDPK |
|  |  |  |  |  | SVENPEAAGGNIFNCVSDR |
|  |  |  |  |  | RPVPIPGSGMQLTNISHVR |
|  |  |  |  |  | DILGWQSTTNLPEDLKER |
|  |  |  |  |  | KRPVPIPGSGMQLTNISHVR |
|  |  |  |  |  | ELLGSGHEVTIFTVGDENSDK |
|  |  |  |  |  | QFLYISSAGIYVPTDEPPHVEGDAVK |
|  |  |  |  |  |  |
| **12** | gi\|255556504 | dihydrolipoamide dehydrogenase | 30 | 1040 | ALGVEYR |
|  |  |  |  |  | MKFMLK |
|  |  |  |  |  | NKVNYVK |
|  |  |  |  |  | GIEGLFKK |
|  |  |  |  |  | TTCIEKR |
|  |  |  |  |  | FPFLANSR |
|  |  |  |  |  | DKAVANLTR |
|  |  |  |  |  | AIDDAEGLVK |
|  |  |  |  |  | ILAEKETDK |
|  |  |  |  |  | MAMASFARR |
|  |  |  |  |  | IGVETDKLGR |
|  |  |  |  |  | VVGVDSSGDGVK |
|  |  |  |  |  | TPFTAGLGLDK |
|  |  |  |  |  | NIIIATGSDVK |
|  |  |  |  |  | SLPGITIDEKK |
|  |  |  |  |  | VGKFPFLANSR |
|  |  |  |  |  | EAAMATHDKPIHI |
|  |  |  |  |  | VCHAHPTMSEALK |
|  |  |  |  |  | IVSSTGALALSEIPK |
|  |  |  |  |  | AEEDGVACVEFIAGK |
|  |  |  |  |  | IVSSTGALALSEIPKK |
|  |  |  |  |  | GTLGGTCLNVGCIPSK |
|  |  |  |  |  | FSSVEVDLPAMMGQK |
|  |  |  |  |  | RGTLGGTCLNVGCIPSK |
|  |  |  |  |  | FISPSEVSVDTLDGGNTVVK |
|  |  |  |  |  | FATNVPGVFAIGDVVPGPMLAHK |
|  |  |  |  |  | LGSEVTVVEFAPDIVPSMDGEIR |
|  |  |  |  |  | LTLEPASGGDQTILEADVVLVSAGR |
|  |  |  |  |  | LGSEVTVVEFAPDIVPSMDGEIRK |
|  |  |  |  |  | HGHVDYDKVPGVVYTHPEVASVGK |
| **13** | gi\|255543455 | glyceraldehyde 3-phosphate dehydrogenase | 21 | 741 | LLDASHR |
|  |  |  |  |  | VAINGFGR |
|  |  |  |  |  | VIQVVSDR |
|  |  |  |  |  | ESADKELK |
|  |  |  |  |  | NPANLPWK |
|  |  |  |  |  | AVALVLPTLK |
|  |  |  |  |  | TFAEEVNAAFR |
|  |  |  |  |  | KTFAEEVNAAFR |
|  |  |  |  |  | VVDLADIVANNWK |
|  |  |  |  |  | GTMTTTHSYTGDQR |
|  |  |  |  |  | DSPLDVIAINDTGGVK |
|  |  |  |  |  | KDSPLDVIAINDTGGVK |
|  |  |  |  |  | TFAEEVNAAFRESADK |
|  |  |  |  |  | VIAWYDNEWGYSQR |
|  |  |  |  |  | GILSVCDEPLVSVDFR |
|  |  |  |  |  | DLGIDLVIEGTGVFVDR |
|  |  |  |  |  | VPTPNVSVVDLVVQVEK |
|  |  |  |  |  | VIQVVSDRNPANLPWK |
|  |  |  |  |  | VPTPNVSVVDLVVQVEKK |
|  |  |  |  |  | YDSTLGIFDADVKPVGTDGISVDGK |
|  |  |  |  |  | GDIPTYVVGVNADGYSADEPIISNASCTTNCLAPFVK |
| **14** | gi\|255585546 | malate dehydrogenase | 16 | 730 | SQASALEK |
|  |  |  |  |  | ALGQISER |
|  |  |  |  |  | NITCLTR |
|  |  |  |  |  | TPSGEKPVK |
|  |  |  |  |  | LNVQVSDVK |
|  |  |  |  |  | EFAPSIPEK |
|  |  |  |  |  | MELVDAAFPLLK |
|  |  |  |  |  | LDSTAEELSEEK |
|  |  |  |  |  | IVQGLSIDEFSR |
|  |  |  |  |  | IVQGLSIDEFSRK |
|  |  |  |  |  | LSSALSAASSACDHIR |
|  |  |  |  |  | KLSSALSAASSACDHIR |
|  |  |  |  |  | VLVTGAAGQIGYALVPMIAR |
|  |  |  |  |  | ELVNDDAWLHGDFISTVQQR |
|  |  |  |  |  | NVIIWGNHSSTQYPDVNHATVK |
|  |  |  |  |  | GVVATTDVVEACTGVNVAVMVGGFPR |
| **15** | gi\|339516172 | ATP synthase CF1 beta subunit | 20 | 626 | IGLFGGAGVGK |
|  |  |  |  |  | LSIFETGIK |
|  |  |  |  |  | SAPAFIQLDTK |
|  |  |  |  |  | VVDLLAPYRR |
|  |  |  |  |  | AVAMSATDGLMR |
|  |  |  |  |  | AHGGVSVFGGVGER |
|  |  |  |  |  | FVQAGSEVSALLGR |
|  |  |  |  |  | VGLTALTMAEYFR |
|  |  |  |  |  | INPTTSGPGVSTLEK |
|  |  |  |  |  | TREGNDLYMEMK |
|  |  |  |  |  | VALVYGQMNEPPGAR |
|  |  |  |  |  | RINPTTSGPGVSTLEK |
|  |  |  |  |  | DVNEQDVLLFIDNIFR |
|  |  |  |  |  | IFNVLGEPVDDLGPVDTR |
|  |  |  |  |  | GMEVIDTGGPLSVPVGGATLGR |
|  |  |  |  |  | MPSAVGYQPTLSTEMGSLQER |
|  |  |  |  |  | DTVGQEINVTCEVQQLLGNNR |
|  |  |  |  |  | GRDTVGQEINVTCEVQQLLGNNR |
|  |  |  |  |  | GIYPAVDPLDSTSTMLQPQIVGEEHYETAQR |
|  |  |  |  |  | EGSITSIQAVYVPADDLTDPAPATTFAHLDATTVLSR |
| **16** | gi\|255544516 | ATP synthase alpha subunit vacuolar | 39 | 1370 | SVWMMR |
|  |  |  |  |  | LIGEIIR |
|  |  |  |  |  | CLGSPER |
|  |  |  |  |  | ALEDETR |
|  |  |  |  |  | ITYSLIK |
|  |  |  |  |  | TPRPVASK |
|  |  |  |  |  | FCPFYK |
|  |  |  |  |  | AREVLQR |
|  |  |  |  |  | LGDLFYR |
|  |  |  |  |  | LASFYER |
|  |  |  |  |  | TVISQALSK |
|  |  |  |  |  | ESEYGYVR |
|  |  |  |  |  | LHDDLTAGFR |
|  |  |  |  |  | LAADTPLLTGQR |
|  |  |  |  |  | VGHDKLIGEIIR |
|  |  |  |  |  | DTVLELEFQGVK |
|  |  |  |  |  | QFTMLQTWPVR |
|  |  |  |  |  | LGDLFYRLVSQK |
|  |  |  |  |  | FEDPAEGEAALVAK |
|  |  |  |  |  | ITYIAPPGQYSLK |
|  |  |  |  |  | EDDLNEIVQLVGK |
|  |  |  |  |  | DTVLELEFQGVKK |
|  |  |  |  |  | TTLVANTSNMPVAAR |
|  |  |  |  |  | DALAEADKITLETAK |
|  |  |  |  |  | EDYLAQNAFTPYDK |
|  |  |  |  |  | DMGYNVSMMADSTSR |
|  |  |  |  |  | YSNSDTVVYVGCGER |
|  |  |  |  |  | EASIYTGITIAEYFR |
|  |  |  |  |  | LAEMPADSGYPAYLAAR |
|  |  |  |  |  | NIIHFYNLANQAVER |
|  |  |  |  |  | LVSQKFEDPAEGEAALVAK |
|  |  |  |  |  | LTTFEDSEKESEYGYVR |
|  |  |  |  |  | LLREDYLAQNAFTPYDK |
|  |  |  |  |  | VSGPVVVADGMAGAAMYELVR |
|  |  |  |  |  | EVLQREDDLNEIVQLVGK |
|  |  |  |  |  | KVSGPVVVADGMAGAAMYELVR |
|  |  |  |  |  | YSSALESFYEQFDPDFINIR |
|  |  |  |  |  | GNEMAEVLMDFPQLTMTLPDGR |
|  |  |  |  |  | LEGDSATIQVYEETAGLTVNDPVLR |
| **17** | gi\|255540797 | betaine-aldehyde dehydrogenase | 14 | 475 | WCKNIK |
|  |  |  |  |  | QLFINGEWR |
|  |  |  |  |  | DWAYTSGAFR |
|  |  |  |  |  | ISDPLEEGCR |
|  |  |  |  |  | LGPVVSGGQYEK |
|  |  |  |  |  | APVSLPMETFK |
|  |  |  |  |  | GKDWAYTSGAFR |
|  |  |  |  |  | SEGATILFGGDRPK |
|  |  |  |  |  | SPIVVFEDVDLDK |
|  |  |  |  |  | QKAPVSLPMETFK |
|  |  |  |  |  | ELGEWGLENYLSVK |
|  |  |  |  |  | LIVHESIANEFVDR |
|  |  |  |  |  | QLFINGEWREPVLK |
|  |  |  |  |  | EVGLPPGVLNILTGLGTEAGAPLASHPR |
| **18** | gi\|255559812 | photosystem II stability/assembly factor HCF136 | 14 | 732 | FNSISFK |
|  |  |  |  |  | IQNMGWR |
|  |  |  |  |  | DGGNTWVSR |
|  |  |  |  |  | RIQNMGWR |
|  |  |  |  |  | GFGILDVGYR |
|  |  |  |  |  | ADGGLWLLVR |
|  |  |  |  |  | SPDGNYVAVSSR |
|  |  |  |  |  | GFVLGNDGVLLR |
|  |  |  |  |  | AAVQETVSATLNR |
|  |  |  |  |  | SIPSAEDEDFNYR |
|  |  |  |  |  | SKDEAWAAGGSGILLR |
|  |  |  |  |  | IPLSAQLPGDMVYIK |
|  |  |  |  |  | GTGISEDFEEISVQSR |
|  |  |  |  |  | TVSSGISGASYYTGTFNTVNR |
| **19** | gi\|255586297 | ferredoxin-NADP reductase | 16 | 512 | LDFAVSR |
|  |  |  |  |  | MYIQTR |
|  |  |  |  |  | LVYTNDK |
|  |  |  |  |  | EEFEKMK |
|  |  |  |  |  | EIAPDNFR |
|  |  |  |  |  | FKPKNPYTGR |
|  |  |  |  |  | MKEIAPDNFR |
|  |  |  |  |  | DNTFVYMCGLK |
|  |  |  |  |  | SFLWKMFFEK |
|  |  |  |  |  | EGQSIGVIPDGIDK |
|  |  |  |  |  | KDNTFVYMCGLK |
|  |  |  |  |  | GIDDIMASLAAKEGK |
|  |  |  |  |  | LYSIASSALGDFGDSK |
|  |  |  |  |  | DPNATIVMLGTGTGIAPFR |
|  |  |  |  |  | GVCSNFLCDLKPGAEVQITGPVGK |
|  |  |  |  |  | ITGDDAPGETWHMVFSTEGEVPYR |
|  |  |  |  |  |  |
| **20** | gi\|255543861 | fructose-bisphosphate aldolase | 14 | 495 | TFEVAQK |
|  |  |  |  |  | EAAWGLAR |
|  |  |  |  |  | EAQEALLIR |
|  |  |  |  |  | TAAYYEQGAR |
|  |  |  |  |  | YTGEGESEEAK |
|  |  |  |  |  | QGMFVKGYVY |
|  |  |  |  |  | ATPQQVADYTLK |
|  |  |  |  |  | LASIGLENTEANR |
|  |  |  |  |  | TVVSIPNGPSALAVK |
|  |  |  |  |  | RLASIGLENTEANR |
|  |  |  |  |  | GILAMDESNATCGKR |
|  |  |  |  |  | MVDVLTEQNIVPGIK |
|  |  |  |  |  | GLVPLPGSNNESWCQGLDGLASR |
|  |  |  |  |  | YAAVSQDNGLVPIVEPEILLDGEHGIDR |
| **21** | gi\|255540493 | elongation factor tu | 17 | 712 | MVMPGDR |
|  |  |  |  |  | EHILLAK |
|  |  |  |  |  | GENQWVDK |
|  |  |  |  |  | FSAIVYVLK |
|  |  |  |  |  | KYDEIDAAPEER |
|  |  |  |  |  | QVGVPNMVVFLNK |
|  |  |  |  |  | VTAIMNDKDEESK |
|  |  |  |  |  | GMVLSKPGTITPHTK |
|  |  |  |  |  | ILDEAMAGDNVGLLLR |
|  |  |  |  |  | HYAHVDCPGHADYVK |
|  |  |  |  |  | GITINTATVEYETENR |
|  |  |  |  |  | MVVELIVPVACEQGMR |
|  |  |  |  |  | KKPHVNIGTIGHVDHGK |
|  |  |  |  |  | HSPFFAGYRPQFYMR |
|  |  |  |  |  | TTLTAALTMALAAMGNSAPK |
|  |  |  |  |  | IYELMDSVDSYIPIPQR |
|  |  |  |  |  | QDQVDDEELLQLVELEVR |
| **22** | gi\|255554879 | ATP synthase gamma chain 2 | 18 | 577 | TRIDSVK |
|  |  |  |  |  | GNSYFIR |
|  |  |  |  |  | TLSIVYNR |
|  |  |  |  |  | KGNSYFIR |
|  |  |  |  |  | RPYIPVDR |
|  |  |  |  |  | VALVVVTGDR |
|  |  |  |  |  | IDSVKNTQK |
|  |  |  |  |  | KTLSIVYNR |
|  |  |  |  |  | KVALVVVTGDR |
|  |  |  |  |  | NTQKITEAMK |
|  |  |  |  |  | FLEGTNLPTAK |
|  |  |  |  |  | GLCGGFNNNIIK |
|  |  |  |  |  | ALQESLASELAAR |
|  |  |  |  |  | DLGVDYTIISVGK |
|  |  |  |  |  | GLCGGFNNNIIKK |
|  |  |  |  |  | DLGVDYTIISVGKK |
|  |  |  |  |  | MSAMSNASDNAAELKK |
|  |  |  |  |  | SDPVIHTLLPLSPEGEICDINGNCVDAAEDEFFR |
| **23** | gi\|255581400 | fructose-bisphosphate aldolase | 14 | 553 | TFEVAQK |
|  |  |  |  |  | EAAWGLAR |
|  |  |  |  |  | AAQEALLIR |
|  |  |  |  |  | WGGRPENLK |
|  |  |  |  |  | TAAYYEQGAR |
|  |  |  |  |  | KWGGRPENLK |
|  |  |  |  |  | YTGEGESEESK |
|  |  |  |  |  | ATPEQVADYTLK |
|  |  |  |  |  | LASIGLENTEANR |
|  |  |  |  |  | RLASIGLENTEANR |
|  |  |  |  |  | GILAMDESNATCGKR |
|  |  |  |  |  | MVDVLVEQNIVPGIK |
|  |  |  |  |  | GLVPLAGSNDESWCQGLDGLASR |
|  |  |  |  |  | YAAISQDSGLVPIVEPEILLDGDHGIDR |
| **24** | gi\|255562761 | oxygen-evolving enhancer protein 1 | 16 | 537 | VPFLFTIK |
|  |  |  |  |  | LTFDEIQSK |
|  |  |  |  |  | NAPPEFQNTK |
|  |  |  |  |  | RLTFDEIQSK |
|  |  |  |  |  | FCLEPTSFTVK |
|  |  |  |  |  | KFCLEPTSFTVK |
|  |  |  |  |  | GGSTGYDNAVALPAGGR |
|  |  |  |  |  | NAPPEFQNTKLMTR |
|  |  |  |  |  | GTGTANQCPTIDGGLDK |
|  |  |  |  |  | DGIDYAAVTVQLPGGER |
|  |  |  |  |  | AESVNKNAPPEFQNTK |
|  |  |  |  |  | GTGTANQCPTIDGGLDKFSFK |
|  |  |  |  |  | LTYTLDEIEGPFEVGPDGTVK |
|  |  |  |  |  | FEEKDGIDYAAVTVQLPGGER |
|  |  |  |  |  | QLVATGKPESFSGEFLVPSYR |
|  |  |  |  |  | GGSTGYDNAVALPAGGRGDEEELAK |
| **25** | gi\|255558698 | cell division protein ftsH | 36 | 1250 | GRTEILK |
|  |  |  |  |  | IVEVLLEK |
|  |  |  |  |  | EIDDSIDR |
|  |  |  |  |  | GTGIGGGNDER |
|  |  |  |  |  | LAEDIDSAIK |
|  |  |  |  |  | ETMTGDEFR |
|  |  |  |  |  | GVLLVGPPGTGK |
|  |  |  |  |  | QVTVDVPDIR |
|  |  |  |  |  | FLEYLDKDR |
|  |  |  |  |  | LAEDIDSAIKR |
|  |  |  |  |  | QVTVDVPDIRGR |
|  |  |  |  |  | ADILDSALLRPGR |
|  |  |  |  |  | QDFMEVVEFLK |
|  |  |  |  |  | VVYGRNHFLSSR |
|  |  |  |  |  | IVAGMEGTVMTDGK |
|  |  |  |  |  | VQLPGLSQELLQK |
|  |  |  |  |  | FDADVSLDIIAMR |
|  |  |  |  |  | LSDSAYEIALSHIR |
|  |  |  |  |  | AILSEFVEIPAENR |
|  |  |  |  |  | KFDADVSLDIIAMR |
|  |  |  |  |  | VRVQLPGLSQELLQK |
|  |  |  |  |  | RLSDSAYEIALSHIR |
|  |  |  |  |  | GLTWFIPADDPTLISK |
|  |  |  |  |  | ADILDSALLRPGRFDR |
|  |  |  |  |  | ENAPCIVFVDEIDAVGR |
|  |  |  |  |  | SSGGMGGPGGPGFPLAFGQSK |
|  |  |  |  |  | RSSGGMGGPGGPGFPLAFGQSK |
|  |  |  |  |  | AKENAPCIVFVDEIDAVGR |
|  |  |  |  |  | TPGFSGADLANLLNEAAILAGR |
|  |  |  |  |  | FQMEPNTGVTFDDVAGVDEAK |
|  |  |  |  |  | VDLFENGTIAIVEAVSPELGNR |
|  |  |  |  |  | KVDLFENGTIAIVEAVSPELGNR |
|  |  |  |  |  | AILSEFVEIPAENRVPPSVSTPVTV |
|  |  |  |  |  | SLVAYHEVGHAICGTLTPGHDAVQK |
|  |  |  |  |  | AAEEVIFGEPEVTTGAAGDLQQITGLAK |
|  |  |  |  |  | EQTLNQLLTEMDGFEGNTGIIVIAATNR |
| **26** | gi\|255582834 | ricin-agglutinin family protein | 13 | 557 | LFSDTTK |
|  |  |  |  |  | FKYIEEK |
|  |  |  |  |  | YTNFMSSLR |
|  |  |  |  |  | LVENFYEDLYPK |
|  |  |  |  |  | SYFFSDVSNEIYGSK |
|  |  |  |  |  | LPYGGSYQALYNAGASR |
|  |  |  |  |  | LEDEDYKPYYVSTVK |
|  |  |  |  |  | KSYFFSDVSNEIYGSK |
|  |  |  |  |  | ARLPYGGSYQALYNAGASR |
|  |  |  |  |  | ENVPLGISQFNNAIFQLVK |
|  |  |  |  |  | YAGAPATTDIASNLVVVIQMISEAAR |
|  |  |  |  |  | GDLLSLENNWGTLSEAIQTSANGNFNPIR |
|  |  |  |  |  | MGLLLAKESMLSIDEEILSWPYNGGLIK |
| **27** | gi\|255550621 | ricin-agglutinin family protein | 15 | 913 | DLSDAIQK |
|  |  |  |  |  | AQVAPSSDQR |
|  |  |  |  |  | GDLVSLENNWK |
|  |  |  |  |  | YATSAATANQAAR |
|  |  |  |  |  | SLLVAVQMVSEAAR |
|  |  |  |  |  | QLESGTTSYSIPVLR |
|  |  |  |  |  | SLLVAVQMVSEAARFK |
|  |  |  |  |  | KQLESGTTSYSIPVLR |
|  |  |  |  |  | LPFGGSYPALENSGAFR |
|  |  |  |  |  | STTMAIDVINAYLVGFK |
|  |  |  |  |  | TKLPFGGSYPALENSGAFR |
|  |  |  |  |  | EQVPLGITQLNDAVFTLYK |
|  |  |  |  |  | YIEQVLVDNFFYLDTER |
|  |  |  |  |  | IGLQDENYQTYYVTTVAEVRPK |
|  |  |  |  |  | VDETNSYYFNDIVNDVYDANPLK |
| **28** | gi\|255549438 | glutathione s-transferase | 13 | 356 | KVYDLGR |
|  |  |  |  |  | DEDLRNK |
|  |  |  |  |  | GIKYEYR |
|  |  |  |  |  | VYGFMLELK |
|  |  |  |  |  | APLFPSDPYSK |
|  |  |  |  |  | VYGFMLELKK |
|  |  |  |  |  | YEYRDEDLR |
|  |  |  |  |  | ESVSASLPDKEK |
|  |  |  |  |  | SALLLQMNPVSK |
|  |  |  |  |  | AQALFWADFVDK |
|  |  |  |  |  | DKAPLFPSDPYSK |
|  |  |  |  |  | AQALFWADFVDKK |
|  |  |  |  |  | NKSALLLQMNPVSK |
| **29** | gi\|255565419 | lactoylglutathione lyase | 10 | 197 | LDFPEMK |
|  |  |  |  |  | TVWTFGQK |
|  |  |  |  |  | RLDFPEMK |
|  |  |  |  |  | GYHNGNSEPR |
|  |  |  |  |  | GYIMQQTMYR |
|  |  |  |  |  | GFGHIGITVDDVYK |
|  |  |  |  |  | SLGVEFVKKPEDGK |
|  |  |  |  |  | ESPANNPGLYTTPDDATK |
|  |  |  |  |  | ATIELTHNWGTESDPDFK |
|  |  |  |  |  | FSLYFMGYENTASAPTDPVER |
| **30** | gi\|255539971 | superoxide dismutase (fe) | 8 | 180 | EQEERR |
|  |  |  |  |  | DFGSFER |
|  |  |  |  |  | AYVDNLNK |
|  |  |  |  |  | LVSWEAVSAR |
|  |  |  |  |  | DTLEYHWGK |
|  |  |  |  |  | LEAAKAQVSER |
|  |  |  |  |  | AQVSEREQEER |
|  |  |  |  |  | RPDYISTFLEK |
| **31** | gi\|255571441 | ferritin | 16 | 461 | ESSEEER |
|  |  |  |  |  | LMQYQNIR |
|  |  |  |  |  | ISEYVAQLR |
|  |  |  |  |  | LLNLHSVADK |
|  |  |  |  |  | KISEYVAQLR |
|  |  |  |  |  | FFKESSEEER |
|  |  |  |  |  | LSSGVSAFSVTTR |
|  |  |  |  |  | MLSSGVSAFSVTTR |
|  |  |  |  |  | ESSEEEREHAEK |
|  |  |  |  |  | GDALYAMELALSLEK |
|  |  |  |  |  | EAFMVPITPQVSLAR |
|  |  |  |  |  | EHAEKLMQYQNIR |
|  |  |  |  |  | SDGVVSPTGNLCSLLQR |
|  |  |  |  |  | LHCIVAPPSEFEHVEK |
|  |  |  |  |  | GHGVWHFDQMLLHEGDAA |
|  |  |  |  |  | NNDPQLADFIESEFLVEQVEDIKK |
| **32** | gi\|255582834 | ricin-agglutinin family protein | 11 | 731 | LFSDTTK |
|  |  |  |  |  | FKYIEEK |
|  |  |  |  |  | YTNFMSSLR |
|  |  |  |  |  | LVENFYEDLYPK |
|  |  |  |  |  | SYFFSDVSNEIYGSK |
|  |  |  |  |  | LPYGGSYQALYNAGASR |
|  |  |  |  |  | LEDEDYKPYYVSTVK |
|  |  |  |  |  | KSYFFSDVSNEIYGSK |
|  |  |  |  |  | ARLPYGGSYQALYNAGASR |
|  |  |  |  |  | ENVPLGISQFNNAIFQLVK |
|  |  |  |  |  | SNVVSYNIPVLPYNLPVTSDNR |
| **33** | gi\|255581166 | major latex protein | 17 | 421 | GHITVLPK |
|  |  |  |  |  | DSTSLFPK |
|  |  |  |  |  | FFPDQYK |
|  |  |  |  |  | IDVVHEAEK |
|  |  |  |  |  | SIEVLEGDGK |
|  |  |  |  |  | IDVVHEAEKK |
|  |  |  |  |  | EVDELIQDQK |
|  |  |  |  |  | SSPDKFWGSIR |
|  |  |  |  |  | EVDELIQDQKV |
|  |  |  |  |  | VSYSVIEGDLLK |
|  |  |  |  |  | ERIDVVHEAEK |
|  |  |  |  |  | LFTYAEGSPIVK |
|  |  |  |  |  | TSDEVEVPHIIK |
|  |  |  |  |  | KVSYSVIEGDLLK |
|  |  |  |  |  | NFKEVDELIQDQK |
|  |  |  |  |  | GDGSLVEWSCEYEK |
|  |  |  |  |  | TSDEVEVPHIIKDFVVK |
| **34** | gi\|255587426 | major latex protein | 11 | 266 | LVVQAIPK |
|  |  |  |  |  | IEADVEIK |
|  |  |  |  |  | FIEGDVMK |
|  |  |  |  |  | DIDAHLAQA |
|  |  |  |  |  | FQTCDLHK |
|  |  |  |  |  | FIEGDVMKEYK |
|  |  |  |  |  | FQTCDLHKGEFGR |
|  |  |  |  |  | GEGSTVHWILEYEK |
|  |  |  |  |  | QIVEDIDDVNMSATYK |
|  |  |  |  |  | FHDVMGGRPHHLTIASPEK |
|  |  |  |  |  | LNADIPDPTSLLEFVVDFTK |
| **35** | gi\|255548059 | hypothetical protein RCOM_1340080 | 3 | 90 | NLVFKPDR |
|  |  |  |  |  | TVQEAAIDR |
|  |  |  |  |  | NLVFKPDREALLEK |
| **36** | gi\|223527364 | cyanate hydratase | 8 | 82 | TEHMVSR |
|  |  |  |  |  | VVVTFDGK |
|  |  |  |  |  | YLPYSEQK |
|  |  |  |  |  | DRVVVTFDGK |
|  |  |  |  |  | MAETKATLTNR |
|  |  |  |  |  | LNEAVMHFGESIK |
|  |  |  |  |  | SYDPNLIQEPSVYR |
|  |  |  |  |  | SYSEIAEETGLTNVYVAQLFR |
| **37** | gi\|255552269 | stem-specific protein TSJT1 | 11 | 463 | QQYGLAK |
|  |  |  |  |  | VEGPAVLAAR |
|  |  |  |  |  | ITSDALVKR |
|  |  |  |  |  | VEGPAVLAARE |
|  |  |  |  |  | SANEVVLVIEAYK |
|  |  |  |  |  | SANEVVLVIEAYKALR |
|  |  |  |  |  | VTAVPAKEEEIWGATFK |
|  |  |  |  |  | LGVFSSAIVSPPDELVAAGSR |
|  |  |  |  |  | SLASFPQGCFYSTAVGELR |
|  |  |  |  |  | SLASFPQGCFYSTAVGELRSFENPK |
|  |  |  |  |  | FLDTNPSAVSLQIGDNAQLAYTHHSESLLQPR |
| **38** | gi\|508716126 | f-box and leucine rich repeat domains containing protein | 34 | 68 | NVALEEK |
|  |  |  |  |  | AQTLEEK |
|  |  |  |  |  | AGKTMFR |
|  |  |  |  |  | LMDEEAK |
|  |  |  |  |  | GGDAHLIR |
|  |  |  |  |  | NLIDCSK |
|  |  |  |  |  | MQLEDSK |
|  |  |  |  |  | WFSDPSN |
|  |  |  |  |  | DERDSSR |
|  |  |  |  |  | NDMMVLR |
|  |  |  |  |  | KEVEQMK |
|  |  |  |  |  | TMFRLHK |
|  |  |  |  |  | SGEKIDFR |
|  |  |  |  |  | LTVSEYDR |
|  |  |  |  |  | TRPTKSGEK |
|  |  |  |  |  | DEGVTHIQK |
|  |  |  |  |  | EELVMKLR |
|  |  |  |  |  | NELHQIKR |
|  |  |  |  |  | ALKLLESYK |
|  |  |  |  |  | IVSGECSDLK |
|  |  |  |  |  | SRNEMEPQK |
|  |  |  |  |  | SKEEENVELR |
|  |  |  |  |  | QSNSHRNQHK |
|  |  |  |  |  | LVKMEGDLTVK |
|  |  |  |  |  | SAIEGEVVAKEK |
|  |  |  |  |  | MELDAEVTEVGK |
|  |  |  |  |  | LFMSIISVENGK |
|  |  |  |  |  | TANDALHQISGLR |
|  |  |  |  |  | ESNDNLSLQLKR |
|  |  |  |  |  | SMAKQTTLEDSSVR |
|  |  |  |  |  | SSMDSDDSAYKILESK |
|  |  |  |  |  | YLHMSLKYAEVEAQR |
|  |  |  |  |  | EDNNYDFHDGSPHAVGVDPVSK |
|  |  |  |  |  | DKEDDMESGVAQNNDALLNIEEEYK |

^a^Spot. is the unique differentially expressed protein spot number.

^b^Database accession numbers according to NCBInr.

^c^The name of the proteins identified by MALDI-TOF/TOF MS.

^d^Number of the matched peptides.

^e^The Mascot searched score against the database NCBInr.
